# The Most Distinctive Causes of Death by State, 2001–2010

**DOI:** 10.5888/pcd12.140395

**Published:** 2015-05-14

**Authors:** Francis P. Boscoe, Eva Pradhan

**Affiliations:** Author Affiliation: Eva Pradhan, MPH, New York State Department of Health, Albany, New York.

**Figure:**
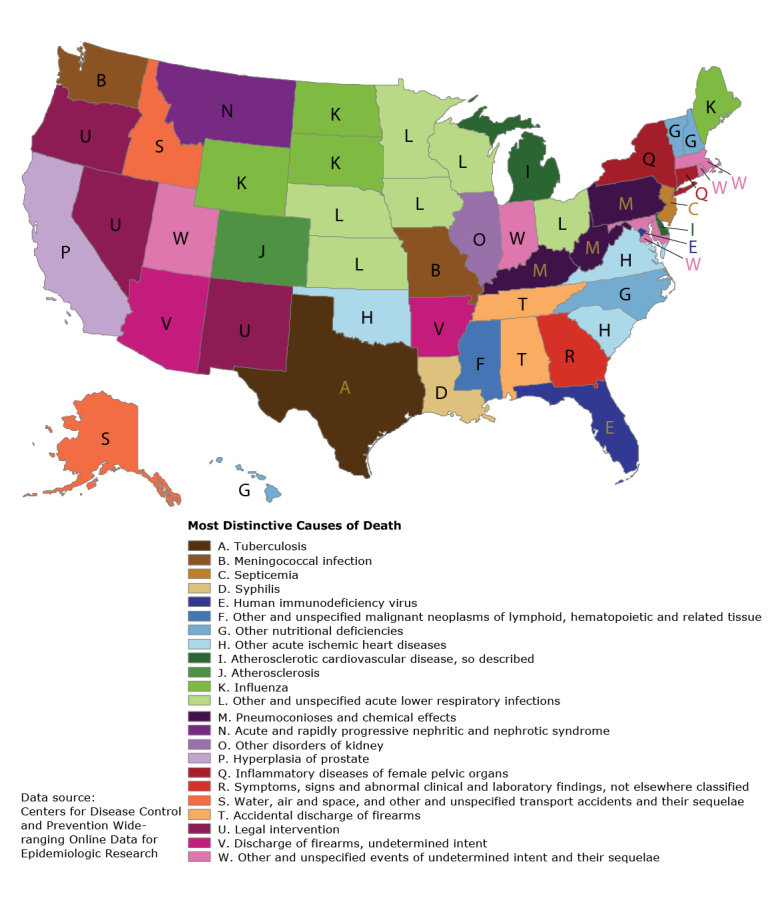
The most distinctive cause of death (defined as the location quotient) for each state and the District of Columbia, 2001–2010. The map shows the cause of death from the *International Classification of Diseases, 10th Revision* (ICD-10), List of 113 Selected Causes of Death with the highest age-adjusted mortality rate ratio in each state. The causes are listed in the legend in the order of disease classification in ICD-10. This map highlights nonstandard cause-of-death certification practices within and between states that can potentially be addressed through education and training.

## Background

Maps of the most distinctive or characteristic value of some variable at the state or country level became popular on social media in 2014. Among the most widely shared examples have been maps of state-level birth name preferences, music-listening preferences, and mortality from among the top 10 causes of death (1). This form of data presentation has a long history in economic geography, where the mapped values are known as location quotients (2). We use the *International Classification of Diseases, 10th Revision* (ICD-10), List of 113 Selected Causes of Death file published by the National Center for Health Statistics (3) to present a more nuanced view of mortality variation within the United States than what can be seen by using only the 10 most common causes of death.

## Methods

Counts for each cause of death included on the ICD-10 List of 113 Selected Causes of Death along with population sizes were obtained for each of the 50 states and the District of Columbia for 2001 through 2010 from the Underlying Cause of Death file accessible through the Centers for Disease Control and Prevention (CDC) WONDER (Wide-ranging Online Data for Epidemiologic Research) website (4). We also included subcauses of death contained in this file, such as specific types of cancer, which brought the total number of causes of death to 136. The standardized mortality rate ratio (ie, the ratio of the age-adjusted state-specific death rate for each cause of death relative to the national age-adjusted death rate for each cause of death, equivalent to a location quotient) was then calculated, and the maximum ratio for each state was mapped. That is, we mapped


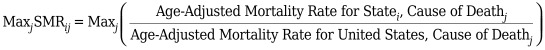


where Max*_j_* is the age-adjusted mortality rate for each state *i* and SMR*_ij_* is the age-adjusted mortality rate for the United States for each cause of death *j*. Causes of death with fewer than 10 counts at the state level were suppressed and therefore not available for this analysis.

The map was produced in SAS software version 9.3 (SAS Institute, Inc) by using a single program that imported the output from CDC WONDER, calculated the mortality rate ratios, and generated the map using PROC MAPIMPORT and PROC GMAP. The program code is available from the authors. Minor cosmetic enhancements were made to the map using Adobe Illustrator (Adobe, Inc). Both colors and numeric labels were used on the map to facilitate black-and-white printing.

## Main Findings

The resulting map depicts a variety of distinctive causes of death based on a wide range of number of deaths, from 15,000 deaths from HIV in Florida to 679 deaths from tuberculosis in Texas to 22 deaths from syphilis in Louisiana. The largest number of deaths mapped were the 37,292 deaths in Michigan from “atherosclerotic cardiovascular disease, so described”; the fewest, the 11 deaths in Montana from “acute and rapidly progressive nephritic and nephrotic syndrome.” The state-specific percentage of total deaths mapped ranged from 1.8% (Delaware; atherosclerotic cardiovascular disease, so described) to 0.0005% (Illinois, other disorders of kidney).

Some of the findings make intuitive sense (influenza in some northern states, pneumoconioses in coal-mining states, air and water accidents in Alaska and Idaho), while the explanations for others are less immediately apparent (septicemia in New Jersey, deaths by legal intervention in 3 Western states). The highly variable use of codes beginning with “other” between states is also apparent. For example, Oklahoma accounted for 24% of the deaths attributable to “other acute ischemic heart diseases” in the country despite having only slightly more than 1% of the population, resulting in a standardized mortality rate ratio of 19.4 for this cause of death, the highest on the map. The highest standardized mortality rate ratio after Oklahoma was 12.4 for pneumoconioses in West Virginia.

A limitation of this map is that it depicts only 1 distinctive cause of death for each state. All of these were significantly higher than the national rate, but there were many others also significantly higher than the national rate that were not mapped. The map is also predisposed to showing rare causes of death — for 22 of the states, the total number of deaths mapped was under 100. Using broader cause-of-death categories or requiring a higher threshold for the number of deaths would result in a different map. These limitations are characteristic of maps generally and are why these maps are best regarded as snapshots and not comprehensive statistical summaries (5).

## Action

This map has been a robust conversation starter among those who have seen it before publication, generating hypotheses and inviting further exploration of the underlying data set, something that an equivalent tabular representation does not accomplish as well. Although chronic disease prevention efforts should continue to emphasize the most common conditions, an outlier map such as this one should also be of interest to public health professionals, particularly insofar as it highlights nonstandard cause-of-death certification practices within and between states that can potentially be addressed through education and training. This is especially true considering that most death certificates are completed by community physicians who receive little or no formal training in this area. For example, a study found that nearly half of the death certificates certified by physicians in a suburban Florida county contained major errors, often reflecting confusion between the underlying cause of death and the terminal mechanism of death (6). It would not take many systematic miscodes involving an unusual cause of death for it to appear on this type of map.
